# Thermal analysis for $A{l}_{2}{O}_{3}$–sodium alginate magnetized Jeffrey’s nanofluid flow past a stretching sheet embedded in a porous medium

**DOI:** 10.1038/s41598-022-06983-1

**Published:** 2022-02-28

**Authors:** Faisal Shahzad, Wasim Jamshed, Kottakkaran Sooppy Nisar, Nor Ain Azeany Mohd Nasir, Rabia Safdar, Abdel-Haleem Abdel-Aty, I. S. Yahia

**Affiliations:** 1 Department of Mathematics, Capital University of Science and Technology (CUST), Islamabad, 44000 Pakistan; 2 Department of Mathematics, College of Arts and Sciences, Prince Sattam Bin Abdulaziz University, Wadi Aldawaser, 11991 Saudi Arabia; 3 Department of Mathematics, Centre for Defence Foundation Studies, Universiti Pertahanan Nasional Malaysia, Kem Sungai Besi, 57000 Kuala Lumpur, Malaysia; 4 Department of Mathematics, Lahore College for Women University, Lahore, 54000 Pakistan; 5 Department of Physics, College of Sciences, University of Bisha, P.O. Box 344, Bisha, 61922 Saudi Arabia; 6 Physics Department, Faculty of Science, Al-Azhar University, Assiut, 71524 Egypt; 7 Laboratory of Nano-Smart Materials for Science and Technology (LNSMST), Department of Physics, Faculty of Science, King Khalid University, P.O. Box 9004, Abha, Saudi Arabia; 8 Research Center for Advanced Materials Science (RCAMS), King Khalid University, P.O. Box 9004, Abha, 61413 Saudi Arabia; 9 Nanoscience Laboratory for Environmental and Biomedical Applications (NLEBA), Semiconductor Lab., Department of Physics, Faculty of Education, Ain Shams University, Roxy, Cairo, 11757 Egypt

**Keywords:** Nanoscience and technology, Physics

## Abstract

The magnetohydrodynamics (MHD) viscous Jeffrey heat transport flow past a permeable extending sheet is analyzed. The Alumina ($A{l}_{2}{O}_{3}$) is chosen as nanoparticles immersed in sodium alginate ($SA$) as the based fluid. The effect of heat generation, Ohmic heating and viscous dissipation are also being investigated adopting Tiwari and Das model. The adequate similarity transformation is used to convert the governing equations to non-linear of higher-order ordinary differential equations (ODEs). The numerical solution of the transformed ODEs is accomplished using a finite-difference technique. The results are described in graphs according to selected parameters’ values provided. The flow velocity reductions when the porosity parameter is augmented. The thermal distribution is affected by the presence of $Pr$, $M$, $\beta$, ${\gamma }^{*}$, ${\delta }^{*}$ and $\phi$. Deborah number and the volume fraction of nanoparticles affect the skin friction coefficient in opposite ways. A higher volume percentage of nanoparticles and a higher Deborah number are both shown to boost the rate of heat transfer. These findings suggest that the concentration of nanoparticles can be used to manipulate heat transport and nanofluid motions.

## Introduction

Before nanotechnology was introduced, the reduced thermal conductivity of several frequently used liquids, such as water, ethylene glycol, kerosene oil, engine oil, has been a challenge for research workers and engineers. The inclusion of a suitable quantity of nanosized particles was proposed by Choi and Eastman^1^ to solve the problem. Choi’s investigations paved the way for advancing and developing techniques to improve conventional fluids’ thermal properties, known as base fluids. Nanofluids keep acquired a lot of consideration in recent years because of their applications^2^ in various fields. This application includes oil recovery, transportation, microfluidics, medical, microelectronics, fuel cells, and manufacturing. Some parameters that can contribute to improving the thermal conductivity exist, such as the Brownian motion effects (see Jang and Choi^3^), as well as nanoparticle size and temperature (see Ref.^4^). Elbashbeshy et al.^5^ have looked at external forces’ contribution and coolant (nanofluid) on rolling cylinders’ mechanical properties. Kameswaran et al.^6^ have undertaken work on magnetohydrodynamic (MHD) nanofluid flows across a stretching and shrinking surface with chemical reaction and viscous expansion. Ganga et al.^7^ studied a nano liquid MHD’s behaviour due to stretching surface with heat generation/absorption effects. Seth and Mishra^8^ have inspected MHD influence on fluid embedded with nanoparticles moving subjected to non-linear expandable surface.

Researchers are currently very interested in everything concerning non-Newtonian liquids. Indeed, these fluids are of considerable importance in various organic and natural, mechanical, and design processes, such as glass design, fibre sheet formation, subsistence items, wire drawing, paper creation, gemstone development, etc. Among the different types of non-Newtonian liquids, Jeffrey fluid model is presented mainly because of a less complicated linear model in which time derivatives are utilized rather than convective derivatives. Xu and Liao^9^ envisioned an analytical solution of time-dependent MHD non-Newtonian flow resulting from an impulse-stretching wall surface. Hung^10^ carried out an analysis of entropy production using non-Newtonian fluid within microchannels and viscous dissipation. Kamali and Binesh^11^ provided a computational study on enhancing the heat transmission using non-Newtonian nanofluids of carbon nanotubes (CNTs) type. They found that the Nusselt number of CNT nanofluid was more remarkable than that of the base fluid. Rundora and Makinde^12^ reviewed how aspiration/injection affects a 3rd-degree fluid with a variable viscosity with an irregular reaction within a channel containing a porous matrix. Sheikholeslami et al.^13^ worked on the MHD flux of nanofluids together with heat transfer and the thermal radiation effect using the Buongiorno model. Eldabe and Abou-zeid^14^ employed the homotopy perturbation technique to solve the magneto-nanofluid flow problem within a porous matrix. Shahsavani et al.^15^ used the experimental results to calculate the heat transfer and pressure fall of nano liquid flow in a cylinder. By the lattice Boltzmann technique, Krishna and Reddy^16^ simulated the magnetohydrodynamic forced convective flow due to stumpy spongy, porous media.

Additionally, boundary layer flows with the stretched surface have numerous applications, including paper production, wire drawing, cooling of a continuous strip, and polymers’ extrusions. Fluid dynamics flow due to an expandable medium was coined by Crane^17^. Bhatnagar et al.^18^ examined non-Newtonian flow produced by a free-flowing stretched sheet. Raptis and Chemical reaction and electromagnetic force were both studied by Perdikis^19^ to see how they affected the flow of viscous fluids across non-linearly stretched surfaces. Thermal and mass transfer parameters of MHD viscous flow generated by porous stretching surfaces were studied by Turkyilmazoglu^20^. Cattaneo–Christov thermal flux impact on viscoelastic MHD fluid flows and heat transfer across a vertical stretching surface were of interest to Li et al.^21^. After that, many studies appear by considering various aspects of the problem^22–29^ to list only a few.

Magnetohydrodynamics refers to the fluid mechanics of the electrically conductive media having an interaction with magnetic fields. Its application in geothermal energy extraction, nuclear reactors, MHD pumps, blood flow measurements, etc. Attia and Kotb^30^ examined the steady-state flow of an incompressible fluid, electrically conductive, flowing between two parallel plates and subjected to an external magnetic force. The effects of thermo-capillary and buoyancy on the movement of an electrical insulator liquid in a rectangular duct when internal heat production is taken into account were studied by Hossain et al.^31^. Malekzadeh et al.^32^ investigate the effects of varying viscosity and radiation on MHD fluid velocity over a moving vertical surface. For the first time, Ghasemi et al.^33^ have quantitatively investigated the effects of sun radiation on MHD flow. An inclined stretched porous sheet with unstable mixed convection of nanofluid flow is studied by Jain et al.^34^. An electrically conducting nanofluid travelling towards an extensible surface was proposed by Nayak et al.^35^. Abel^36^ scrutinized the effect of viscous dissipation on fluid past an extending surface and found that fluid temperature amplifies in the case of enrichment in the heat dissipation parameter. Kishan and Deepa^37^ contemplated micropolar fluid flow along an expandable sheet having pores embedded with heat dissipation, stagnation point as well as viscous dissipation effects. Alim et al.^38^ pondered Newtonian-based fluid embedded with results like Ohmic and viscous dissipation over a vertical expandable sheet surface. Ferdows et al.^39^ were interested in viscous dissipation effects associated with Hall current on liquid flow through an expanding medium.

A Casson fluid is a shear thinning liquid with an infinite viscosity at zero rate of shear and a zero viscosity at an infinite rate of shear^40^. Casson fluid is commonly found in honey, jelly, soup, tomato sauce, concentrated fruit liquids, and other foods. It is also the best rheological model for blood and chocolate^41^. Casson fluid also has yield stress and is very important in polymer processing industries and biomechanics^42^. The Jeffrey fluid model can represent the stress relaxation property of non-Newtonian fluids, whereas the traditional viscous fluid model cannot. The Jeffrey fluid model well describes a class of non-Newtonian fluids with the distinctive memory time scale, also known as the relaxation time^43^. Relaxation time is the period of time when a system relaxes in response to changes in external variables^44,45^. It refers to the time it takes for a polymer coil to relax from a distorted condition to its equilibrium configuration. It is an important parameter in determining the properties of a viscoelastic fluid. Non-Newtonian fluids include drilling muds, apple sauce, foams, soaps, sugar solution pastes, clay coating, ketchup, lubricant, some oils, colloidal and suspension solutions. Non-Newtonian fluids are classified into three types: differential, integral, and rate.

The goal of the analysis is to assess the boundary-layer $A{l}_{2}{O}_{3}$–sodium alginate magnetized viscoelastic Jeffrey’s nanofluid flow due to a continuous surface. Moreover, this type of work for the non-Newtonian fluid model is even more narrow down. This work is primarily concerned with investigating the heat transfer flow of Jeffrey nanomaterial across a flexible surface. For the following reasons, the present investigation has a discernible novelty in it. (i) the incorporation of the $A{l}_{2}{O}_{3}$ mono nanoparticles with sodium alginate-based fluid, (ii) the embodiment of magnetohydrodynamics effect, (iii) the analysis of the heat transfer flow in the light of the Ohmic heating, viscous heat dissipation, porous medium and heat source and (iv) the utilization of Tiwari and Das^46^ model with the thermophysical attributes of nanofluids. The modified ODEs were solved digitally using MATLAB software and the finite difference method. The stimulus of dimensionless factors on velocity and heat fields was studied in detail with graphs. In addition, the impact of various dimensionless factors on drag coefficient and local Nusselt number was investigated.

## Mathematical modelling

**Figure 1:**
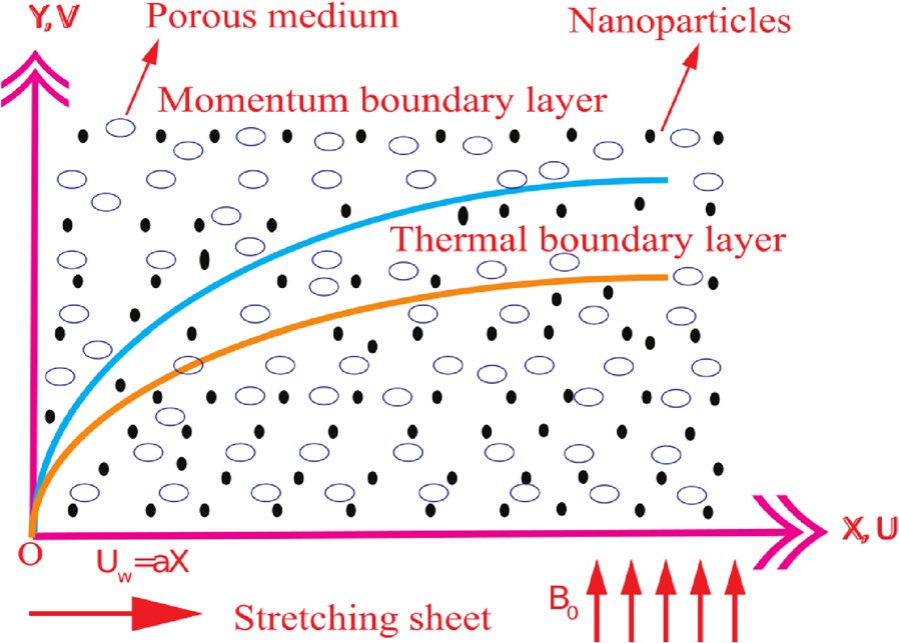
Physical interpretation of flow geometry.

We deliberate an $A{l}_{2}{O}_{3}$–sodium alginate Jeffrey nanofluid laminarly and electrically conducting flow through a stretchable sheet. The nanofluid experiencing a magnetic field ${B}_{0}$ employed in the $Y$-axis direction, and flow is bounded in $Y>0$, as presented in Fig. 1. The stretchable sheet is along the $X$-axis path, and the flow is known as linearly stretching with velocity ${U}_{w}=aX$ such that $a$ is a positive constant. The plate wall temperature is known as ${{\yen }}_{w}$ and assumed to have a quadratic type expansion at $Y=0$, i.e., ${{\yen }}_{w}={{\yen }}_{\infty }+A{\left(\frac{X}{L}\right)}^{2}$ to administer the nanofluid flow. The induced magnetic field impact is ignored, which is justifiable in the small magnetic Reynolds number.

Subject to these assumptions, the boundary layer equations administering the nanofluid flow and the heat fields can be presented in the dimensional form given below

1 $$\frac{\partial U}{\partial X} + \frac{\partial V}{\partial Y}=0,$$

2 $$\left.\begin{array}{ll}& (U\frac{\partial U}{\partial X}+V\frac{\partial U}{\partial Y})=\frac{{\nu }_{nf}{\lambda }_{2}}{(1+{\lambda }_{1})}(U\frac{{\partial }^{3}U}{\partial X{\partial }^{2}Y}- \frac{\partial U}{\partial X}\frac{{\partial }^{2}U}{\partial {Y}^{2}}+ \frac{\partial U}{\partial Y}\frac{{\partial }^{2}U}{\partial X\partial Y} + V\frac{{\partial }^{3}U}{\partial {Y}^{3}})\\ & \frac{{\nu }_{nf}}{(1+{\lambda }_{1})}\frac{{\partial }^{2}U}{\partial {Y}^{2}}-\frac{{\sigma }_{nf}}{{\rho }_{nf}}{B}_{0}^{2}U-\frac{{\mu }_{nf}}{{\rho }_{nf}}\frac{U}{K},\end{array}\right\}$$

3 $$U\frac{\partial {\yen }}{\partial X} +V\frac{\partial {\yen}}{\partial Y}= \frac{{k}_{nf}}{(\rho {c}_{p}{)}_{nf}} \frac{{\partial }^{2} {\yen}}{\partial {Y}^{2}} + \frac{{\mu }_{nf}}{(\rho {c}_{p}{)}_{nf}}(\frac{\partial U}{\partial Y}{)}^{2} + \frac{{\sigma }_{nf}}{(\rho {c}_{p}{)}_{nf}}{B}_{0}^{2}{U}^{2} + \frac{{Q}_{0}}{(\rho {c}_{p}{)}_{nf}}( {\yen}-{ {\yen}}_{\infty }).$$

The corresponding endpoint conditions are:

4 $$\left.\begin{array}{ll}& Y=0: U={U}_{w}, V=0, {\yen}={ {\yen}}_{w},\\ & Y \to \infty :U \to 0, \frac{\partial U}{\partial Y} \to 0, {\yen} \to { {\yen}}_{\infty }.\end{array}\right\}$$

The thermophysical characteristics of nanofluids are described as follows (see Tables 1 and 2)^46,47^:

**Table 1:** Thermophysical characteristics of Jeffrey nanofluid.

Properties	Nanofluid
Dynamic viscosity $(\mu )$	${\mu }_{nf}$ = ${\mu }_{f}(1-\phi {)}^{-2.5}$
Density $(\rho )$	${\rho }_{nf}$ = $(1-\phi ){\rho }_{f}$ + $\phi {\rho }_{s}$
Heat capacity $(\rho {C}_{p})$	$(\rho {C}_{p}{)}_{nf}$ = $(1-\phi )(\rho {C}_{p}{)}_{f}$ + $\phi (\rho {C}_{p}{)}_{s}$
Electrical Conductivity $(\sigma )$	$\frac{{\sigma }_{nf }}{{\sigma }_{f }}=1+\frac{3({\sigma }_{s}-{\sigma }_{f })\phi }{({\sigma }_{s }+2{\sigma }_{f })-({\sigma }_{s }-{\sigma }_{f })\phi }$
Thermal Conductivity $(\kappa )$	$\frac{{\kappa }_{nf}}{{\kappa }_{f}}$ = $\left[\frac{({\kappa }_{s}+2{\kappa }_{f})-2\phi ({\kappa }_{f}-{\kappa }_{s})}{({\kappa }_{s}+2{\kappa }_{f})+\phi ({\kappa }_{f}-{\kappa }_{s})}\right]$

**Table 2:** Values of thermophysical features^48^.

Thermophysical	$\rho (\mathrm{kg}/{\mathrm{m}}^{3})$	${c}_{p} (\mathrm{J}/\mathrm{kgK})$	$k(\mathrm{W}/\mathrm{mK})$	$\sigma (\mathrm{S}/\mathrm{m})$	Pr
Sodium alginate $(SA)$	989	4175	0.6376	$2.6\times 1{0}^{-4}$	6.5
Alumina $(A{l}_{2}{O}_{3})$	3970	765.0	40.000	$2.7\times 1{0}^{-1}$	–

Initiate the following similarity variables, which transform Eqs. (1)–(3) into the ODEs

5 $$\psi =-\sqrt{a\nu } X F(\varsigma ), \theta =\frac{ {\yen}- { {\yen}}_{\infty }}{{ {\yen}}_{w}- { {\yen}}_{\infty }}, \varsigma =\sqrt{\frac{a}{\nu }}Y,$$

and stream function $\psi$ is expressed as follows

6 $$U=\frac{\partial \psi }{\partial Y} \mathrm{and} V=-\frac{\partial \psi }{\partial X}.$$

So we have

7 $$U = a X {F}^{^{\prime}}(\varsigma ), V=-\sqrt{a\nu }F(\varsigma ).$$

Employing Eqs. (5) and (7), Eqs. (1)–(3) are turned into

8 $$\left.\begin{array}{ll}& {F}^{{^{\prime}}{^{\prime}}{^{\prime}}}-\frac{{K}_{2}}{{K}_{1}}(1+{\lambda }_{ 1}) \left[({F}^{^{\prime}}{)}^{2} - F {F}^{{^{\prime}}{^{\prime}}}\right]+\beta \left[( {F}^{{^{\prime}}{^{\prime}}}{)}^{2}-F{F}^{iv}\right]-(1+{\lambda }_{1})\frac{{K}_{5 }}{{K}_{1}}M{F}^{^{\prime}}\\ & -(1+{\lambda }_{1})\frac{1}{{K}_{1}}{\gamma }^{*}{F}^{^{\prime}}=0,\end{array}\right\}$$

9 $${\theta }^{{^{\prime}}{^{\prime}}} +\frac{{K}_{3}}{{K}_{4}}Pr(F{\theta }^{^{\prime}}-2\theta {F}^{^{\prime}})+\frac{{K}_{1}}{{K}_{4}}PrEc{F}^{{^{\prime}}{^{\prime}}}+\frac{1}{{K}_{4}}EcPrM({F}^{^{\prime}}{)}^{2}+\frac{1}{{K}_{4}}Pr{\delta }^{*}\theta =0,$$

subjected to BCs

10 $$\left.\begin{array}{ll}& \varsigma =0: F(0)=0, {F}^{^{\prime}}(0)=1, \theta (0)=1\\ & \varsigma \to \infty : {F}^{^{\prime}}(\varsigma ) \to 0, \theta (\varsigma ) \to 0.\end{array}\right\}$$

Various dimensionless parameters occurring in Eqs. (8) and (9) are delineated as

11 $$\left.\begin{array}{l}\beta =a{\lambda }_{2} (\text{Deborah number}), M=\frac{{\sigma }_{f}{B}_{0}^{2}}{a{\rho }_{f}} (\text{MHD parameter}), \\ {\gamma }^{*}=\frac{{\nu }_{nf}}{aK} (\text{porosity parameter}), {\delta }^{*}=\frac{{Q}_{0}}{(a\rho {c}_{p}{)}_{nf}} (\text{heat source}/\text{sink}), \\ Pr=\frac{{\mu }_{f}({c}_{p}{)}_{f}}{{k}_{f}} (\text{Prandtl number}), Ec=\frac{{a}^{2}{l}^{2}}{({{ {\yen}}_{w}}-{{ {\yen}}_{\infty }})({c}_{p}{)}_{f}} (\text{Eckert number}), \\ {K}_{1}=(1-{\phi }_{SA}{)}^{-2.5}, {K}_{2}=[(1-{\phi }_{SA})+\phi \frac{{\rho }_{s}}{{\rho }_{f}}], {K}_{3}=[(1-{\phi }_{SA})+{\phi }_{SA} \frac{(\rho {c}_{p}{)}_{s}}{(\rho {c}_{p}{)}_{f}}],\\ {K}_{4}= \frac{({k}_{s}+2 {k}_{f})-2 {\phi }_{SA} ({k}_{f}-{k}_{s})}{\left({k}_{s}+2 {k}_{f}\right)-({k}_{f}-{k}_{s}){\phi }_{SA}} {K}_{5}= 1+\frac{3({\sigma }_{s}-{\sigma }_{f }){\phi }_{SA}}{({\sigma }_{s }+2{\sigma }_{f })-({\sigma }_{s }-{\sigma }_{f }){\phi }_{SA}}.\end{array}\right\}$$

The expressions regarding substantial surface drag ${C}_{f}$ and heat transfer $N{u}_{X}$, are manifested by

12 $${C}_{f} = \frac{2 {\tau }_{w}}{{\rho }_{f}{U}_{w}^{2}}, N{u}_{X}=\frac{X{q}_{w}}{{k}_{f}({ {\yen}}_{w}-{ {\yen}}_{\infty })},$$

expressions regarding shear as well as heat flux at the wall are manifested by ${\tau }_{w} = \frac{{\mu }_{nf}}{\left(1+{\lambda }_{1}\right)}\left[\frac{\partial U}{\partial Y}+{\lambda }_{2}\left(U\frac{{\partial }^{2}U}{\partial Y\partial X}+V\frac{{\partial }^{2}U}{{\partial Y}^{2}}\right)\right]$ and ${q}_{w}=-{k}_{nf}(\frac{\partial {\yen}}{\partial Y})$ respectively and moreover Eq. (12) can be transformed likewise

13 $$\left.\begin{array}{ll}& {C}_{f} R{e}^{1/2} = \frac{\left(1+\beta \right)}{\left(1+{\lambda }_{1}\right)(1-\phi {)}^{2.5}}{F}^{{^{\prime}}{^{\prime}}}(0),\\ & R{e}_{X}^{-1/2} N{u}_{X} = -\frac{{\kappa }_{nf}}{{\kappa }_{f}}{\theta }^{^{\prime}}(0),\end{array}\right\}$$

whereas the expression regarding Reynolds number is $R{e}_{X}=\frac{{U}_{X}}{{\nu }_{f}}$.

## Solution methodology

**Figure 2:**
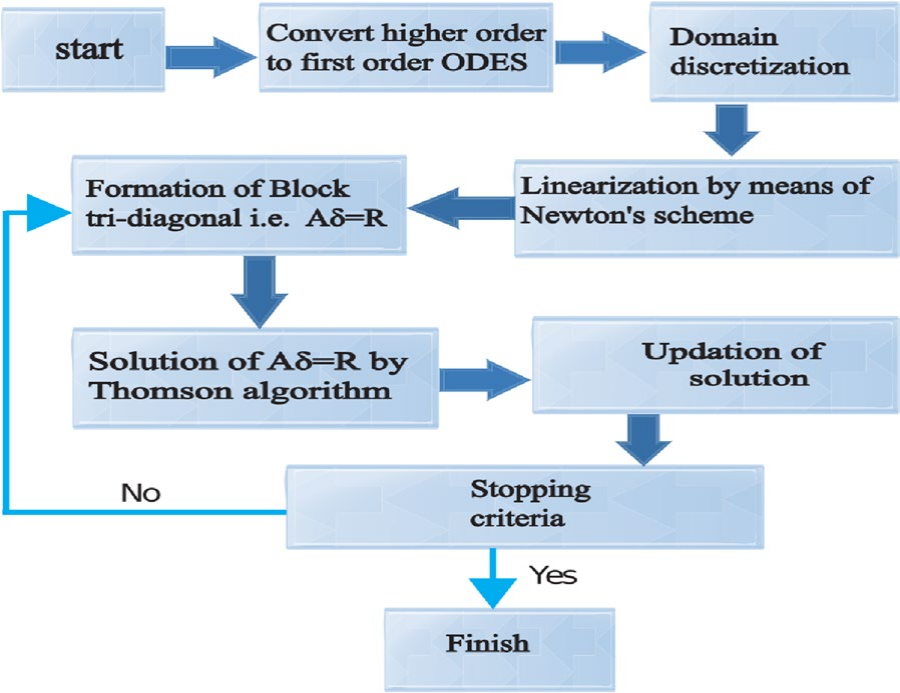
Flow chart illustrating the Keller box method.

The dimensionless ODEs (8)–(9) along with Eq. (10) can be solved using a reliable numerical technique named the Keller box method^49,50^ with the aid of MATLAB software. The flow chart (see Fig. 2) mechanisms of this scheme are displayed below to achieve numerical outcomes:

## Numerical procedure

We introduce dependent variables $\Gamma \tilde{u }$, $\Gamma \tilde{v }$, $\Gamma \tilde{w }$ and $\Gamma \tilde{t }$ such that

14 $$\frac{dF}{d\varsigma }=\Gamma \tilde{u }, \frac{d\Gamma \tilde{u }}{d\varsigma }=\Gamma \tilde{v }, \frac{d\Gamma \tilde{v }}{d\varsigma }=\Gamma \tilde{w }, \frac{d\theta }{d\varsigma }=\Gamma \tilde{t }.$$

So that Eqs. (8) and (9) can be written as

15 $$-\beta F\frac{d\Gamma \tilde{w }}{d\varsigma }+\Gamma \tilde{w }-\frac{{K}_{2}}{{K}_{1}}(1+{\lambda }_{1})[\Gamma {\tilde{u }}^{2}-F\Gamma \tilde{v }]+\beta\Gamma {\tilde{v }}^{2}-\frac{{K}_{5}}{{K}_{1}}(1+{\lambda }_{1})(M+{\gamma }^{*})\Gamma \tilde{u }=0$$

and

16 $$\frac{d\Gamma \tilde{t }}{d\varsigma }+\frac{{K}_{3}}{{K}_{4}}Pr(F\Gamma \tilde{t }-2\Gamma \tilde{u }\theta )+\frac{{K}_{1}}{{K}_{4}}PrEc\Gamma {\tilde{v }}^{2}+\frac{1}{{K}_{4}}MPrEc\Gamma {\tilde{u }}^{2}+\frac{1}{{K}_{4}}Pr{\delta }^{*}\theta =0,$$

dimensionless BCs are enumerated by

17 $$\left.\begin{array}{ll}& F(0)=0,\Gamma \tilde{u }(0)=1,\quad \theta (0)=1,\\ &\Gamma \tilde{u }\to 0, \theta \to 0\, \mathrm{as}\quad \varsigma \to \infty .\end{array}\right\}$$

The domain of the system can be discretized with the following nodes:

${\varsigma }_{0}=0, {\varsigma }_{j}={\varsigma }_{j-1}+{h}_{j}, j=\mathrm{0,1},\mathrm{2,3}...,J, {\varsigma }_{J}={\varsigma }_{\infty },$whereas the term ${h}_{j}$ indicates step-size. Equations (14)–(16) with the central utilization difference can be approximated at the midpoint ${\varsigma }_{j-1/2}$, likewise

18 $$\frac{{F}_{j}-{F}_{j-1}}{{h}_{j}}=\frac{\Gamma {\tilde{u }}_{j}+\Gamma {\tilde{u }}_{j-1}}{2},$$

19 $$\frac{\Gamma {\tilde{u }}_{j}-\Gamma {\tilde{u }}_{j-1}}{{h}_{j}}=\frac{\Gamma {\tilde{v }}_{j}+\Gamma {\tilde{v }}_{j-1}}{2},$$

20 $$\frac{\Gamma {\tilde{v }}_{j}-\Gamma {\tilde{v }}_{j-1}}{{h}_{j}}=\frac{\Gamma {\tilde{w }}_{j}+\Gamma {\tilde{w }}_{j-1}}{2},$$

21 $$\frac{{\theta }_{j}-{\theta }_{j-1}}{{h}_{j}}=\frac{\Gamma {\tilde{t }}_{j}+\Gamma {\tilde{t }}_{j-1}}{2},$$

22 $$\left.\begin{array}{l}-\frac{{K}_{2}}{{K}_{1}}(1+{\lambda }_{ 1})[(\frac{\Gamma {\tilde{u }}_{j }+\Gamma {\tilde{u }}_{j-1 }}{2}{)}^{2}-(\frac{{F}_{j}+{F}_{j-1}}{2})(\frac{\Gamma {\tilde{v }}_{j }+\Gamma {\tilde{v }}_{j-1 }}{2})]+\\ \frac{\Gamma {\tilde{w }}_{j}+\Gamma {\tilde{w }}_{j-1 }}{2}+\beta [(\frac{\Gamma {\tilde{v }}_{j }+\Gamma {\tilde{v }}_{j-1 }}{2}{)}^{2}-(\frac{{F}_{j}+{F}_{j-1}}{2})(\frac{\Gamma {\tilde{w }}_{j}-\Gamma {\tilde{w }}_{j-1}}{{h}_{j}})]\\ -\frac{{K}_{5}}{{K}_{1}}(M+{\gamma }^{*})(1+{\lambda }_{1})(\frac{\Gamma {\tilde{u }}_{j}+\Gamma {\tilde{u }}_{j-1}}{2})=0\end{array}\right\},$$

23 $$\left.\begin{array}{l}\frac{{K}_{3}}{{K}_{4}}Pr(\frac{{F}_{j}+{F}_{j-1}}{2})(\frac{\Gamma {\tilde{t }}_{j}+\Gamma {\tilde{t }}_{j-1}}{2})-2\frac{{K}_{3}}{{K}_{4}}Pr(\frac{\Gamma {\tilde{u }}_{j}+\Gamma {\tilde{u }}_{j-1}}{2})(\frac{{\theta }_{j}+{\theta }_{j-1}}{2})+\\ +\frac{\Gamma {\tilde{t }}_{j}-\Gamma {\tilde{t }}_{j-1}}{{h}_{j}}+\frac{{A}_{1 }}{{A}_{5 }}PrEc(\frac{\Gamma {\tilde{v }}_{j }+\Gamma {\tilde{v }}_{j-1 }}{2}{)}^{2}+\frac{{A}_{3 }}{{A}_{5 }}MPrEc(\frac{\Gamma {\tilde{u }}_{j }+\Gamma {\tilde{u }}_{j-1 }}{2}{)}^{2}\\ +\frac{1}{{K}_{4}}Pr{\delta }^{*}(\frac{{\theta }_{j}+{\theta }_{j-1}}{2})=0.\end{array}\right\}$$

Equations (18)–(23) can be further linearized with the help of a well-known scheme called Newton’s method by introducing the substitutions mentioned underneath:

24 $$\left.\begin{array}{ll}& {F}_{j}^{n+1}={F}_{j}^{n}+\delta {F}_{j}^{n},\Gamma {\tilde{u }}_{j}^{n+1}=\Gamma {\tilde{u }}_{j}^{n}+\delta\Gamma {\tilde{u }}_{j}^{n},\Gamma {\tilde{v }}_{j}^{n+1}=\Gamma {\tilde{v }}_{j}^{n}+\delta\Gamma {\tilde{v }}_{j}^{n},\\ &\Gamma {\tilde{w }}_{j}^{n+1}=\Gamma {\tilde{w }}_{j}^{n}+\delta\Gamma {\tilde{w }}_{j}^{n},\Gamma {\tilde{t }}_{j}^{n+1}=\Gamma {\tilde{t }}_{j}^{n}+\delta\Gamma {\tilde{t }}_{j}^{n}, {\theta }_{j}^{n+1}={\theta }_{j}^{n}+\delta {\theta }_{j}^{n}.\end{array}\right\}$$

Putting these expressions in (18)–(23) and dropping terms having higher powers in terms of $\delta$ to get the system of equation mentioned below

25 $$\delta {F}_{j }-\delta {F}_{j-1 }-\frac{{h}_{j }}{2}(\delta\Gamma {\tilde{u }}_{j }+\delta\Gamma {\tilde{u }}_{j-1 })=({r}_{1}{)}_{j },$$

26 $$\delta\Gamma {\tilde{u }}_{j }-\delta\Gamma {\tilde{u }}_{j-1 }-\frac{{h}_{j}}{2}(\delta \tilde{v }{ }_{j}+\delta \tilde{v }{ }_{j-1})=({r}_{2}{)}_{j},$$

27 $$\delta\Gamma {\tilde{v }}_{j }-\delta\Gamma {\tilde{v }}_{j-1 }-\frac{{h}_{j}}{2}(\delta\Gamma {\tilde{w }}_{j }+\delta\Gamma {\tilde{w }}_{j-1 })=({r}_{3}{)}_{j },$$

28 $$\delta {\theta }_{j }-\delta {\theta }_{j-1 }-\frac{{h}_{j}}{2}(\delta\Gamma {\tilde{t }}_{j }+\delta\Gamma {\tilde{t }}_{j-1 })=({r}_{4}{)}_{j },$$

29 $$\begin{aligned} & ({\xi }_{1}{)}_{j}\delta\Gamma {\tilde{w }}_{j}+({\xi }_{2}{)}_{j}\delta\Gamma {\tilde{w }}_{j-1}+({\xi }_{3}{)}_{j}\delta {F}_{j}+({\xi }_{4}{)}_{j}\delta {F}_{j-1}+({\xi }_{5}{)}_{j}\delta\Gamma {\tilde{v }}_{j} \\ & \quad +({\xi }_{6}{)}_{j}\delta\Gamma {\tilde{v }}_{j-1}+({\xi }_{7}{)}_{j}\delta\Gamma {\tilde{u }}_{j}+({\xi }_{8}{)}_{j}\delta\Gamma {\tilde{u }}_{j-1}=({r}_{5}{)}_{j}, \end{aligned}$$

30 $$\begin{aligned} & \quad ({\epsilon }_{1}{)}_{j}\delta\Gamma {\tilde{t }}_{j}+({\epsilon }_{2}{)}_{j}\delta\Gamma {\tilde{t }}_{j-1}+({\epsilon }_{3}{)}_{j}\delta {F}_{j}+({\epsilon }_{4}{)}_{j}\delta {F}_{j-1}+({\epsilon }_{5}{)}_{j}\delta\Gamma {\tilde{u }}_{j}+({\xi }_{6}{)}_{j}\delta\Gamma {\tilde{u }}_{j-1} \\ & \quad +({\epsilon }_{7}{)}_{j}\delta {\theta }_{j}+({\epsilon }_{8}{)}_{j}\delta {\theta }_{j-1}+({\epsilon }_{9}{)}_{j}\delta\Gamma {\tilde{v }}_{j}+({\epsilon }_{10}{)}_{j}\delta\Gamma {\tilde{v }}_{j-1}=({r}_{6}{)}_{j},\end{aligned}$$

where

31 $$\left.\begin{array}{ll} ({\xi }_{1}{)}_{j}&=-\frac{\beta }{2}({F}_{j}+{F}_{j-1})+\frac{{h}_{j}}{2}, ({\xi }_{2}{)}_{j}=\frac{\beta }{2}({F}_{j}+{F}_{j-1})+\frac{{h}_{j}}{2},\\ ({\xi }_{3}{)}_{j}&=-\frac{\beta }{2}(\Gamma {\tilde{w }}_{j}+\Gamma {\tilde{w }}_{j-1})+\frac{{K}_{2}}{{K}_{1}}\frac{{h}_{j}}{4}(1+{\lambda }_{1})(\Gamma {\tilde{v }}_{j}+\Gamma {\tilde{v }}_{j-1})=({\xi }_{4}{)}_{j},\\ ({\xi }_{5}{)}_{j}&=\frac{{K}_{2}}{{K}_{1}}\frac{{h}_{j}(1+{\lambda }_{1})({F}_{j}+{F}_{j-1})}{4}+\frac{\beta {h}_{j}(\Gamma {\tilde{v }}_{j}+\Gamma {\tilde{v }}_{j-1})}{2}=({\xi }_{6}{)}_{j},\\ ({\xi }_{7}{)}_{j}&=-\frac{{K}_{2}}{{K}_{1}}\frac{{h}_{j}(1+{\lambda }_{1})(\Gamma {\tilde{u }}_{j}+\Gamma {\tilde{u }}_{j-1})}{2}-\frac{1}{{K}_{1}}\frac{(M+{\gamma }^{*}){h}_{j}(1+{\lambda }_{1})}{2}=({\xi }_{8}{)}_{j},\\ ({r}_{5}{)}_{j}&=\frac{(\Gamma {\tilde{w }}_{j}+\Gamma {\tilde{w }}_{j-1})}{2}(\beta ({F}_{j}+{F}_{j-1})-{h}_{j})\\ & \quad-\frac{{K}_{2}}{{K}_{1}}\frac{{h}_{j}(1+{\lambda }_{1})({F}_{j}+{F}_{j-1})(\Gamma {\tilde{v }}_{j}+\Gamma {\tilde{v }}_{j-1})}{4}\\ & \quad-\frac{\beta {h}_{j}(\Gamma {\tilde{v }}_{j}+\Gamma {\tilde{v }}_{j-1}{)}^{2}}{4}+\frac{{K}_{2}}{{K}_{1}}\frac{{h}_{j}(1+{\lambda }_{1})(\Gamma {\tilde{u }}_{j}+\Gamma {\tilde{u }}_{j-1}{)}^{2}}{4}\\ & \quad+\frac{{K}_{5}}{{K}_{1}}\frac{(M+{\gamma }^{*}){h}_{j}(1+{\lambda }_{1})(\Gamma {\tilde{u }}_{j}+\Gamma {\tilde{u }}_{j-1})}{2},\end{array}\right\}$$

32 $$\left.\begin{array}{ll} ({\epsilon }_{1}{)}_{j}&=1+\frac{{K}_{3}}{{K}_{4}}\frac{Pr{h}_{j}({F}_{j}+{F}_{j-1})}{4}, ({\epsilon }_{2}{)}_{j}=({\epsilon }_{1}{)}_{j}-2,\\ ({\epsilon }_{3}{)}_{j}&=\frac{{K}_{3}}{{K}_{4}}\frac{Pr{h}_{j}(\Gamma {\tilde{t }}_{j}+\Gamma {\tilde{t }}_{j-1})}{4}=({\epsilon }_{4}{)}_{j},\\ ({\epsilon }_{5}{)}_{j}&=-\frac{{K}_{3}}{{K}_{4}}\frac{Pr{h}_{j}({\theta }_{j}+{\theta }_{j-1})}{2}+\frac{1}{{K}_{4}}\frac{MPrEc{h}_{j}(\Gamma {\tilde{u }}_{j}+\Gamma {\tilde{u }}_{j-1})}{2}=({\epsilon }_{6}{)}_{j},\\ ({\epsilon }_{7}{)}_{j}&=\frac{1}{{K}_{4}}\frac{Pr{h}_{j}{\delta }^{*}}{2}-\frac{{K}_{3}}{{K}_{4}}\frac{Pr{h}_{j}(\Gamma {\tilde{u }}_{j}+\Gamma {\tilde{u }}_{j-1})}{2}=({\epsilon }_{8}{)}_{j},\\ ({\epsilon }_{9}{)}_{j}&=\frac{{K}_{1}}{{K}_{4}}\frac{PrEc{h}_{j}(\Gamma {\tilde{v }}_{j}+\Gamma {\tilde{v }}_{j-1})}{2}=({\epsilon }_{10}{)}_{j},\\ ({r}_{6}{)}_{j}&=-\frac{{K}_{3}}{{K}_{4}}\frac{Pr{h}_{j}({F}_{j}+{F}_{j-1})(\Gamma {\tilde{t }}_{j}+\Gamma {\tilde{t }}_{j-1})}{4}-\frac{1}{{K}_{4}}\frac{Pr{h}_{j}{\delta }^{*}({\theta }_{j}+{\theta }_{j-1})}{2}\\ & \quad+(\Gamma {\tilde{t }}_{j-1}-\Gamma {\tilde{t }}_{j})-\frac{{K}_{1}}{{K}_{4}}\frac{PrEc{h}_{j}(\Gamma {\tilde{v }}_{j}+\Gamma {\tilde{v }}_{j-1}{)}^{2}}{4}-\frac{1}{{K}_{4}}\frac{MPrEc{h}_{j}(\Gamma {\tilde{u }}_{j}+\Gamma {\tilde{u }}_{j-1}{)}^{2}}{4}.\end{array}\right\}$$

After the linearizing process, the tridiagonal block matrix mentioned below is achieved.

33 $$A\delta =R,$$

where

$$A = \left[ {\begin{array}{*{20}l} {\left[ {~A_{{1~}} } \right]} \hfill & {\left[ {C_{{1~}} } \right]} \hfill & {} \hfill & {} \hfill & {} \hfill & {} \hfill & {} \hfill \\ {} \hfill & {\left[ {~A_{{2~}} } \right]} \hfill & {\left[ {~C_{{2~}} } \right]} \hfill & {} \hfill & {} \hfill & {} \hfill & {} \hfill \\ {} \hfill & {} \hfill & {} \hfill & \ddots \hfill & {} \hfill & {} \hfill & {} \hfill \\ {} \hfill & {} \hfill & {} \hfill & \ddots \hfill & {} \hfill & {} \hfill & {} \hfill \\ {} \hfill & {} \hfill & {} \hfill & \ddots \hfill & {} \hfill & {} \hfill & {} \hfill \\ {} \hfill & {} \hfill & {} \hfill & {} \hfill & {\left[ {B_{{J - 1~}} } \right]} \hfill & {\left[ {~A_{{J - 1~}} } \right]} \hfill & {\left[ {C_{{J - 1~}} } \right]} \hfill \\ {} \hfill & {} \hfill & {} \hfill & {} \hfill & {} \hfill & {\left[ {B_{{J~}} } \right]} \hfill & {\left[ {A_{{J~}} } \right]} \hfill \\ \end{array} } \right],~\delta = \left[ {\begin{array}{*{20}l} {\left[ {\delta _{1} } \right]} \hfill \\ {} \hfill \\ \vdots \hfill \\ \vdots \hfill \\ \vdots \hfill \\ {\left[ {\delta _{{J - 1}} } \right]} \hfill \\ {\left[ {\delta _{J} } \right]} \hfill \\ \end{array} } \right]{\text{and}}~~R = \left[ {\begin{array}{*{20}l} {\left[ {~R_{1} } \right]} \hfill \\ {} \hfill \\ \vdots \hfill \\ \vdots \hfill \\ \vdots \hfill \\ {\left[ {~R_{{J - 1}} } \right]} \hfill \\ {\left[ {~R_{J} } \right]} \hfill \\ \end{array} } \right].$$

where the elements defined in Eq. (33) are

$$\left[\begin{array}{l}{A}_{1 }\end{array}\right]=\left[\begin{array}{llllll}0& 0& 0& 1& 0& 0\\ -0.5{h}_{1}& 0& 0& 0& 0& 0\\ -1& -0.5{h}_{1}& 0& 0& -0.5{h}_{1}& 0\\ 0& 0& -0.5{h}_{1}& 0& 0& -0.5{h}_{1}\\ ({\xi }_{6}{)}_{1}& ({\xi }_{2}{)}_{1}& 0& ({\xi }_{3}{)}_{1}& ({\xi }_{1}{)}_{1}& 0\\ ({\epsilon }_{10}{)}_{1}& 0& ({\epsilon }_{2}{)}_{1}& ({\epsilon }_{3}{)}_{1}& 0& ({\epsilon }_{1}{)}_{1}\\ & & & & & \end{array}\right],$$

$$\left[\begin{array}{l}{A}_{j}\end{array}\right]=\left[\begin{array}{llllll}-0.5{h}_{j}& 0& 0& 1& 0& 0\\ -1& -0.5{h}_{j}& 0& 0& 0& 0\\ 0& -1& 0& 0& -0.5{h}_{j}& 0\\ 0& 0& -1& 0& 0& -0.5{h}_{j}\\ ({\xi }_{8}{)}_{j}& ({\xi }_{6}{)}_{j}& 0& ({\xi }_{3}{)}_{j}& ({\xi }_{1}{)}_{j}& 0\\ ({\epsilon }_{6}{)}_{j}& ({\epsilon }_{10}{)}_{j}& ({\epsilon }_{8}{)}_{j}& ({\epsilon }_{3}{)}_{j}& 0& ({\epsilon }_{1}{)}_{j}\\ & & & & & \end{array}\right],2\le j\le J$$

$$\left[\begin{array}{l}{B}_{j}\end{array}\right]=\left[\begin{array}{llllll}0& 0& 0& -1& 0& 0\\ 0& 0& 0& 0& 0& 0\\ 0& 0& 0& 0& -0.5{h}_{j}& 0\\ 0& 0& 0& 0& 0& -0.5{h}_{j}\\ 0& 0& 0& ({\xi }_{4}{)}_{j}& ({\xi }_{2}{)}_{j}& 0\\ 0& 0& 0& ({\epsilon }_{4}{)}_{j}& 0& ({\epsilon }_{2}{)}_{j}\\ & & & & & \end{array}\right],2\le j\le J$$

$$\left[\begin{array}{l}{C}_{j}\end{array}\right]=\left[\begin{array}{lllllll}-0.5{h}_{j}& 0& 0& 0& 0& 0& \\ 1& -0.5{h}_{j}& 0& 0& 0& 0& \\ 0& 1& 0& 0& 0& 0& \\ 0& 0& 1& 0& 0& 0& 0\\ ({\xi }_{7}{)}_{j}& ({\xi }_{5}{)}_{j}& 0& 0& 0& 0& \\ ({\epsilon }_{5}{)}_{j}& ({\epsilon }_{9}{)}_{j}& ({\epsilon }_{7}{)}_{j}& 0& 0& 0& \\ & & & & & & \end{array}\right],1\le j\le J-1.$$

Now we factorize A as

34 $$A=LU,$$

where

$$L=\left[\begin{array}{llllll}[{\alpha }_{1 }]& & & & & \\ & [ {\alpha }_{2 }]& & & & \\ & & & \ddots & & \\ & & & \ddots & [ {\alpha }_{J-1}]& \\ & & & & [{B}_{J }]& [{\alpha }_{J }]\end{array}\right],U=\left[\begin{array}{llllll}[I]& [ {\Gamma }_{1 }]& & & & \\ & [I]& [ {\Gamma }_{2 }]& & & \\ & & \ddots & \ddots & & \\ & & & [I]& [ {\Gamma }_{J-1 }]& \\ & & & & [I]& \end{array}\right],$$

wherein A is a tridiagonal block structure of order $J\times J$ with each block having a size of $6\times 6$, and $[I]$ is a unit block of order 6. Equation (33) can be tackled using the LU factorization approach to give numerical results of $\delta$. Because the physical domain of the issue is unbounded, but the computing domain must be limited, we apply far-field boundary constraints so ${\varsigma }_{max}=10$. In order to compute the numerical results, the step size and error tolerance level can be adjusted at ${h}_{j}=0.01$ and $1{0}^{-5}$. A healthy comparison with previously published research might be used to assess the credibility of forthcoming outcomes of Hayat et al.^51^ and Ishak et al.^52^ in the case of ${\theta }^{^{\prime}}(0)$ by retaining $\beta =\phi =Ec=M=0$ (Table 3). Table 3 further compares the bvp4c MATLAB code and KBM solutions for various embedding parameter values for ${\theta }^{^{\prime}}(0)$. A comparison analysis reveals a high level of agreement. In the case of $\phi =0$ makes the problem identical to that of Ahmad et al.^53^.

**Table 3:** Nusselt number ${\theta }^{^{\prime}}(0)$ comparison by keeping $\phi =\beta =0.0$.

$Pr$	Hayat et al.^51^	Ishak et al.^52^	bvp4c code	Present study
0.5	0.72325	0.72323	0.72320	0.72327
5	3.16243	3.16242	3.16235	3.16249
10	4.64681	4.64682	4.64679	4.64686
15	5.78369	5.78367	5.78368	5.78373

## Results and discussions

**Figure 3:**
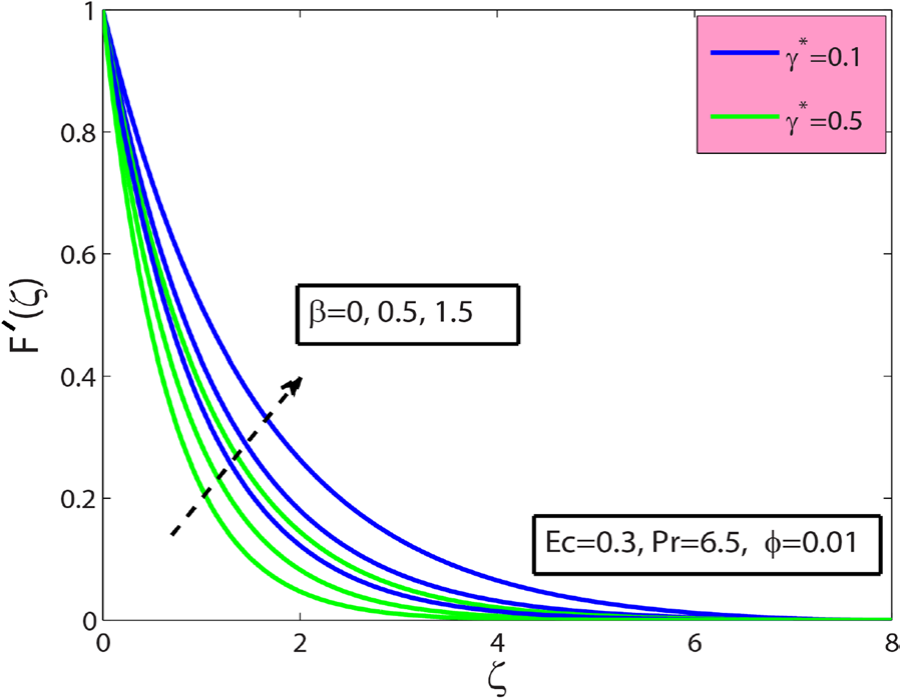
Sway of $\beta$ $\&$ ${\gamma }^{*}$ on $F(\zeta )$.

**Figure 4:**
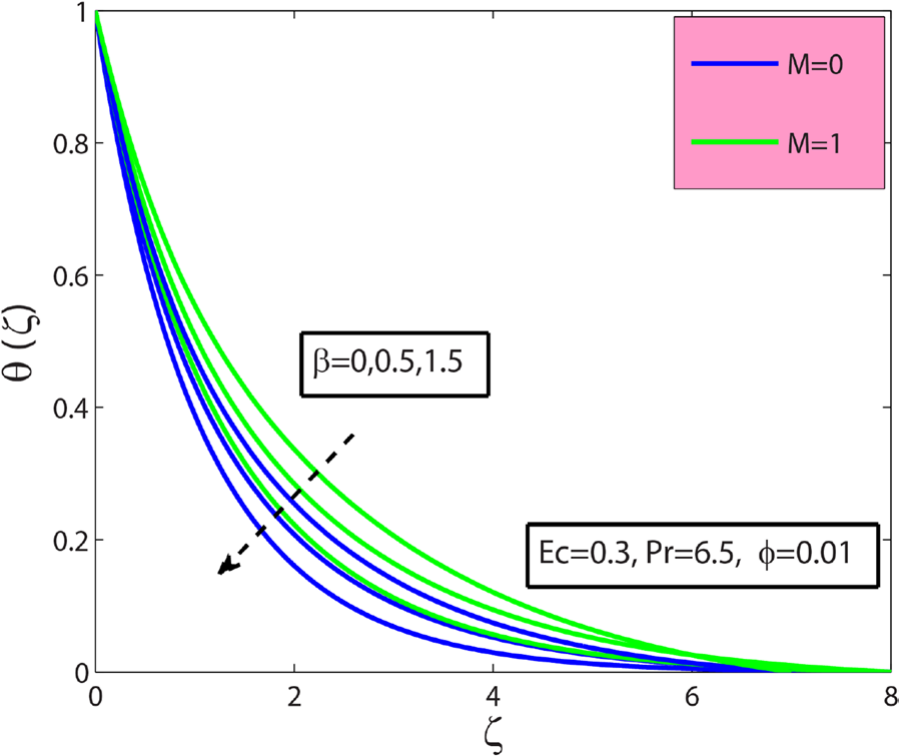
Sway of $\beta$ $\&$ $M$ on $\theta (\zeta )$.

**Figure 5:**
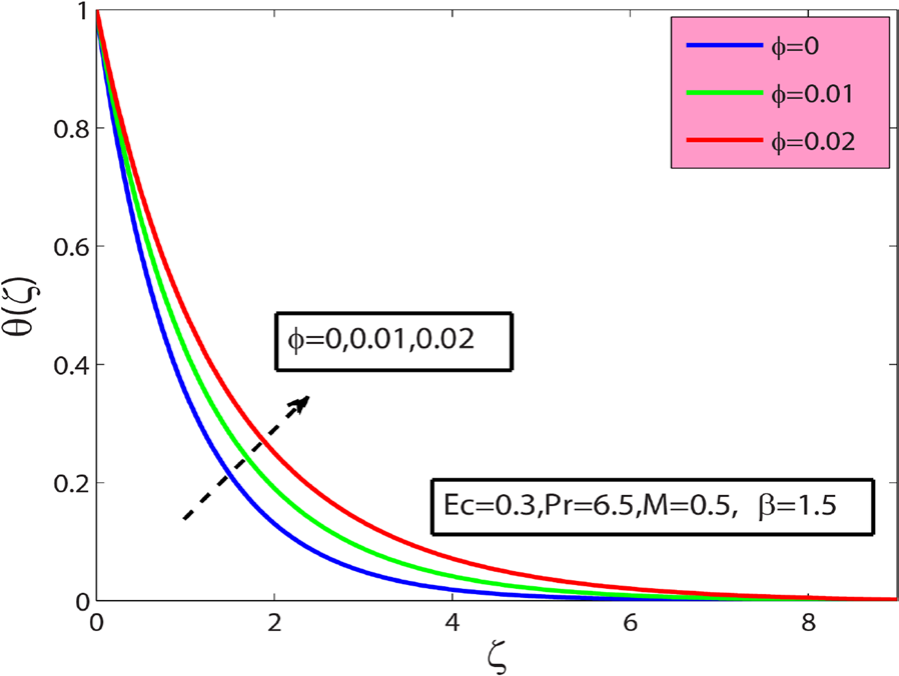
Sway of $\phi$ on $\theta (\zeta )$.

**Figure 6:**
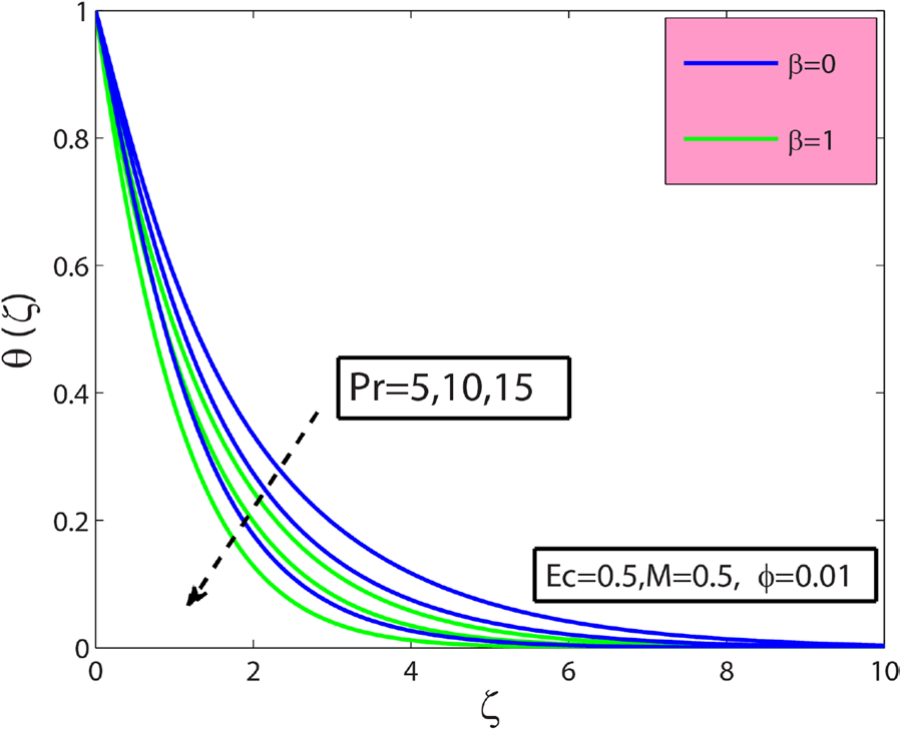
Sway of $\beta$ $\&$ $Pr$ on $\theta (\zeta )$.

**Figure 7:**
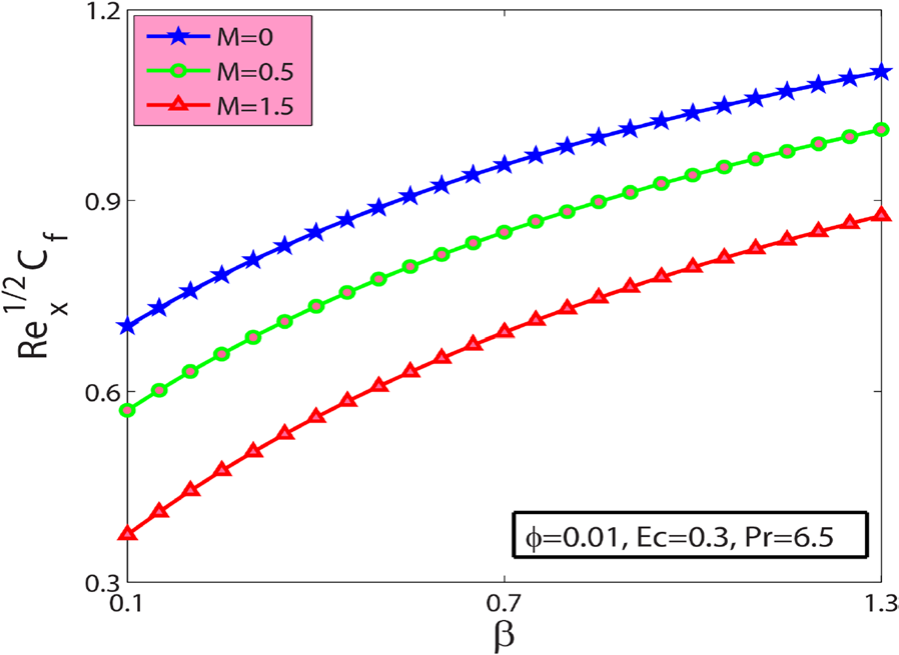
Sway of $\beta$ $\&$ $M$ on the skin friction coefficient.

**Figure 8:**
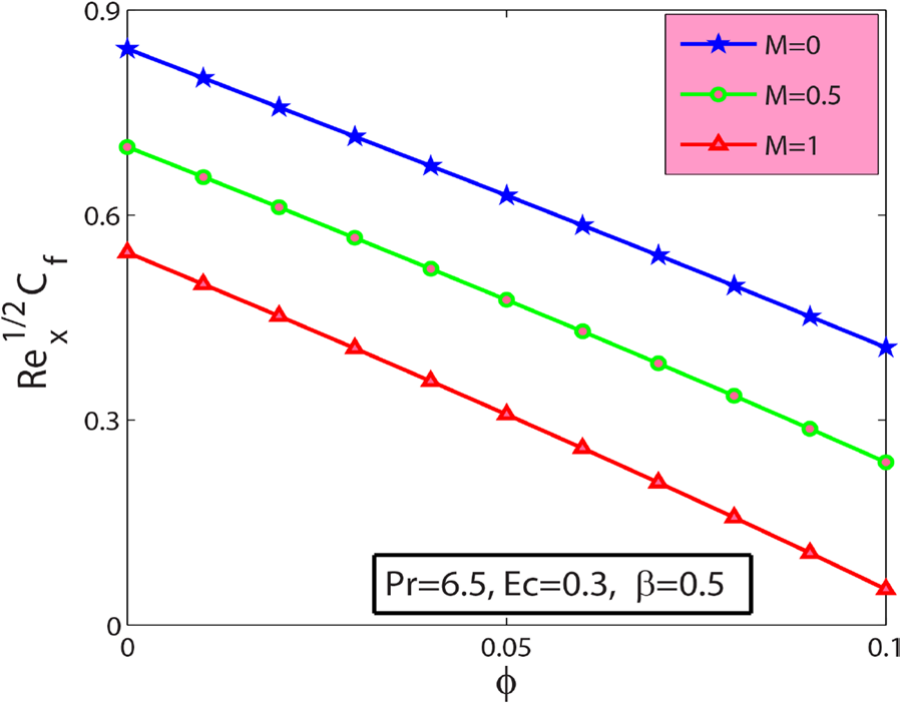
Sway of $\phi$ $\&$ $M$ on the skin friction coefficient.

This research’s primary goal is to explore nanofluids’ flow and heat transfer characteristics due to a stretching sheet. The values of surface drag and heat transfer in terms of $A{l}_{2}{O}_{3}$–sodium alginate nanofluid are computed and displayed in Table 4. Magnification in $\phi$ and magnetic term $M$ depreciates the surface drag effect but elevates for the case of positive variation in Deborah number $\beta$. Amplification in $\phi$, $\beta$, and Prandtl number $\mathrm{Pr}$ amplifies heat transfer phenomenon while depreciates for the case of magnification in $M$, and Eckert number $Ec$. In the present section, the physical significance of sundry parameters such as Deborah number $\beta (0\le \beta \le 1.5)$, magnetic parameter $M(0\le M\le 1)$, porosity parameter ${\gamma }^{*}(0.1\le {\gamma }^{*}\le 0.5)$, nanoparticle volume fraction $\phi (0\le \phi \le 0.1)$, Prandtl number $Pr(10\le Pr\le 20)$ and Eckert number $Ec(0.3\le Ec\le 5)$ versus velocity, temperature and heat transfer rate is examined through Figs. 3, 4, 5, 6, 7 and 8. The field ${f}^{^{\prime}}(\varsigma )$ is noticeably enhanced for the rising $\beta$ and this outcome is in the augmentation of momentum boundary layer thickness. As $\beta$ depends upon the retardation time ${\lambda }_{2}$, higher ${\lambda }_{2}$ offers rise to the fluid flow, because of which the velocity field is elevated. The effect of ${\gamma }^{*}$ is monitored from Fig. 3, and it is discovered that the existence of ${\gamma }^{*}$ diminishes the thickness of the boundary layer as well as the velocity field. This phenomenon happens because an increase in the permeability parameter increases the resistance to fluid motion, resulting in a reduction in velocity. This occurrence reflects in slower fluidity over the surface for $A{l}_{2}{O}_{3}-SA$ Jeffery nanofluid in comparison with that of base fluid may be due to added particles making the nanofluid combo flow slower. Figure 4 displays $\beta$ effect on the thermal field $\theta (\varsigma )$. Here, the thermal field is a decreasing function of $\beta$ because $\beta$ causes a decrease in the molecular movements, which eventually decreases the fluid’s temperature profile. From Fig. 4, it is pretty clear that magnification in $M$ amplifies the temperature profile. More significant values of $M$ mean a rise in Lorentz force that is a resistive force. It generates a kind of friction on the flow field; more heat energy is caused due to this friction. Hence thermal profile enhanced. Figure 5 shows that the nanoparticle concentration $\phi$ increases the fluid temperature. Physically, increasing $\phi$ brings about an enhancement in the thermal conductivity phenomenon, which elevates the thermal profile. Figure 6 reflects the performance of Prandtl number $\mathrm{Pr}$ on the temperature field for both viscous nanofluid ($\beta =0$) and Jeffrey nanofluid ($\beta =1$), respectively. It can be noted from the figure that the fluid temperature reduces with escalating $\mathrm{Pr}$. This incidence is because of a decrement in $\mathrm{Pr}$ amplifying thermal conductivities. Hence heat can diffuse away from the heated surface more quickly than larger values of $\mathrm{Pr}$. Consequently, in the case of the lesser $\mathrm{Pr}$, the heat transfer rate is reduced. It is observed that the introduction of $\beta$ reduces the thermal profile. The fluctuation of the local skin friction coefficient with β for various values of M is seen in Fig. 7. It has been found that when the value of β increases, the skin friction coefficient increases as well. This prevalence is because an increase causes more fluid particles to move into the boundary layer, particularly in the region of the sheet surface. As a result, the velocity boundary layer thickness decreases, resulting in greater skin friction coefficient values. On the other hand, the skin friction coefficient falls as M increases. The magnetic field has the effect of slowing down the velocity. As a result, the magnetic field may be employed to regulate flow properties.

**Table 4:** Numerical outcomes for ${C}_{f}R{e}_{x}^{1/2}$ and $N{u}_{X}R{e}_{X}^{-1/2}$ for diverse values of sundry dimensionless parameters.

$\phi$	$\beta$	$M$	$Pr$	$Ec$	${C}_{f}R{e}_{x}^{1/2}$	$N{u}_{X}R{e}_{X}^{-1/2}$
0	0.5	0.5	6.5	0.3	0.209005	3.226895
0.01					0.198873	3.328853
0.02					0.199823	3.417300
0.01	0.0	0.2	6.5	0.3	0.339209	1.473574
	0.5				0.433021	1.596740
	1				0.507677	1.693113
0.01	0.2	0.0	6.5	0.3	0.412718	4.933286
		0.5			0.359445	4.363074
		1			0.308883	3.811261
0.01	0.5	0.5	5	0.3	0.197353	0.993617
			10		0.197353	3.672235
			15		0.197353	4.195064
0.01	2	0.5	6.5	0.5	0.575514	1.554988
				1	0.575514	1.118344
				1.5	0.575514	0.681700

**Figure 9:**
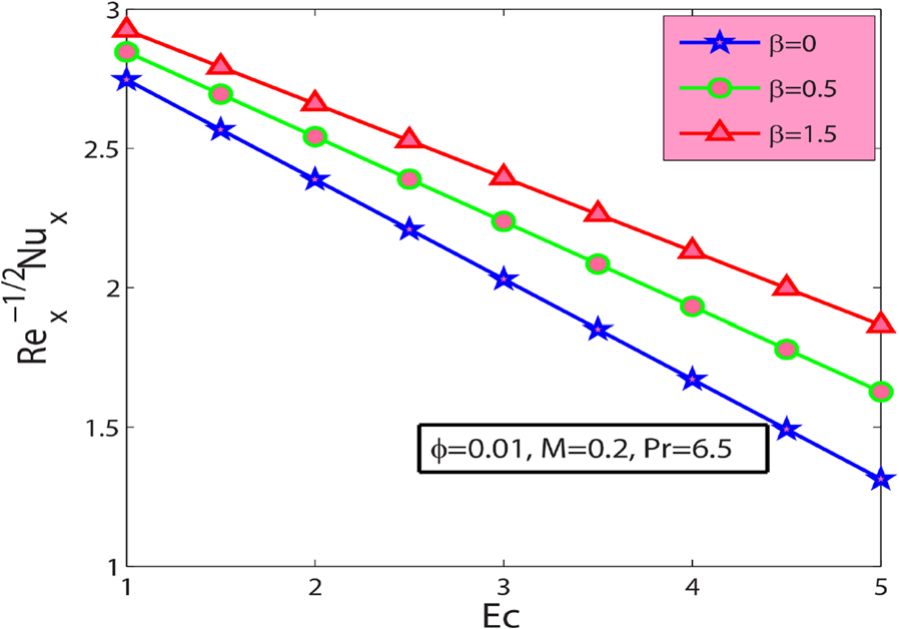
Sway of $\beta$ $\&$ $Ec$ on $-{\theta }^{^{\prime}}(0)$.

**Figure 10:**
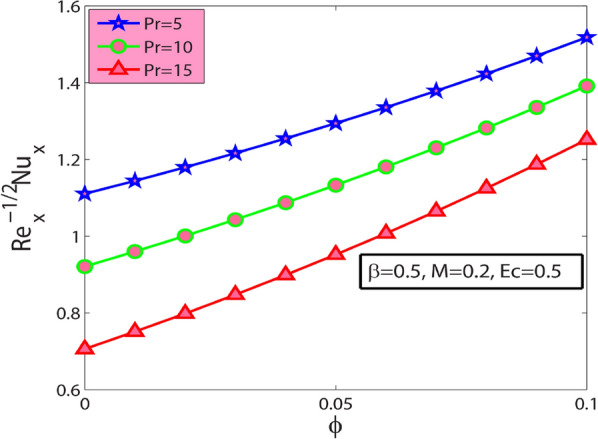
Sway of $\phi$ $\&$ $Pr$ on $-{\theta }^{^{\prime}}(0)$.

It is noticed from Fig. 8 that the local skin friction coefficient decreases with increase the magnitude of volume fraction of nanoparticles ф. Figure 9 depicts the heat transfer rate $-{\theta }^{^{\prime}}(0)$ with $Ec$ for diverse values of $\beta$. It shows that the heat transport rate at the wall enhances with Deborah number $\beta$ and declines with Eckert number parameter $Ec$. It can be noticed from Fig. 10 that heat transfer rate $-{\theta }^{^{\prime}}(0)$ amplify with a rise in $\phi$. Also, it is identified from Fig. 10 that the heat transfer rate $-{\theta }^{^{\prime}}(0)$ reduces for rising values of $\mathrm{Pr}$. Physically, thermal conductivity is reduced for a more excellent value of Pr. Consequently, their heat conduction ability lessens. Heat transport rate abates near the wall.

## Concluding remarks

The numerical solutions of MHD Jeffery nanofluid flow and heat transfer past over a stretching sheet with Joule heating and viscous dissipation effects are investigated. The calculated solutions are validated by comparing them to previously published results. The following are some of the findings that have been explored and summarised:

- Flow field declines for rising the values of porosity parameter.
- Thermal profile climbs with a boost in the concentration of nanoparticles and Lorentz force.
- The interaction of Deborah number is proven to be counterproductive in embellishing temperature distribution though salubrious in accomplishing higher velocity inside flow field.
- The thermal profile is abbreviating function of the Prandtl number.
- Nanoparticles volume fraction $\phi$ supports the heat transfer rate but tends to reduce the skin frictions of the $A{l}_{2}{O}_{3}$–sodium alginate nanofluid. Independent study on heat transfer rate shows that the parameters like Deborah number $\beta$ favour the Nusselt numbers while the Prandtl number $Pr$ and Eckert number $Ec$ tend to oppose it.
- $A{l}_{2}{O}_{3}$–Sodium alginate nanofluid possesses a small drag coefficient and heat transfer rate compared to the base fluid.
- By and large, $A{l}_{2}{O}_{3}$–Sodium alginate Jeffrey nanofluid combinations carry an upper hand in the main aspects of heat transfer efficiency compared to the sodium alginate base fluid.

## Nomenclature

$$U,V \left[{\mathrm{m\;s}}^{-1}\right]$$: Velocity components
$${c}_{p}$$: Specific heat
${C}_{f}$: Skin friction coefficient
$${\yen}_{w}\left[\mathrm{K}\right]$$: Temperature
$${f}^{^{\prime}}(\varsigma )$$: Dimensionless velocity
$${\yen}_{\infty } \left[\mathrm{K}\right]$$: Environmental temperature
${C}_{f}$: Surface drag coefficient
$$L\left[\mathrm{m}\right]$$: Sheet length
${B}_{0}\left[{\mathrm{A\;m}}^{-1}\right]$: Magnetic field strength
$${U}_{w}\left[{\mathrm{m\;s}}^{-1}\right]$$: Stretching speed
$$a\left[{\mathrm{s}}^{-1}\right]$$: Stretching rate
$${\gamma }^{*}$$: Porosity parameter
$$Nu$$: Nusselt number
${q}_{w}\left[{\mathrm{W\;m}}^{-1}\;{\mathrm{K}}^{-1}\right]$: Heat flux at the wall
$$\nu \left[{\mathrm{m}}^{2}{\mathrm{s}}^{-1}\right]$$: Kinematic viscosity
$k\left[{\mathrm{m\;s}}^{-1}\right]$: Thermal-based conductivity
$${\yen }\left[K\right]$$: Temperature
$$s$$: Solid phase
$$f$$: Fluid phase
$$nf$$: Nanofluid
$\rho \left[{\mathrm{kg\;m}}^{-3}\right]$: Density of fluid
${\lambda }_{1}$: Relaxation to retardation times
$${\lambda }_{2}$$: Retardation time
$\phi$: Volume fraction of nanoparticles
$$\varsigma$$: Similarity variable
$K$: Porous medium permeability
$$\theta$$: Dimensionless temperature
$$\psi$$: Stream function
${\delta }^{*}$: Heat source/sink
$$\alpha \left[{\mathrm{m}}^{2}\;{\mathrm{s}}^{-1}\right]$$: Thermal diffusivity
$$\mu \left[{\mathrm{kg\;m}}^{-1}\;{\mathrm{s}}^{-1}\right]$$: Dynamic viscosity
$\sigma \left[{\mathrm{Sm}}^{-1}\right]$: Electric-based conductivity
${\tau }_{w}\left[{\mathrm{Nm}}^{-2}\right]$: Wall shear stress
$$\beta$$: Deborah number
